# Using sequence analysis to visualize exposure to pregnancy in the postpartum period

**DOI:** 10.4054/demres.2025.53.1

**Published:** 2025-07-01

**Authors:** Dana Sarnak, Linnea Zimmerman, Wenxuan Huang, Alison Gemmill

**Affiliations:** 1 Department of Population, Family and Reproductive Health, Johns Hopkins Bloomberg School of Public Health, 615 North Wolfe Street, Baltimore, MD 21205 USA.; 2 Department of Sociology, Johns Hopkins University, 533 Mergenthaler Hall, 3400 North Charles Street, Baltimore, MD 21218 USA.

## Abstract

**BACKGROUND:**

Exposure to pregnancy during the postpartum period is shaped by biological and behavioral determinants, such as resumption of sexual activity, return of menses, and contraceptive use dynamics.

**OBJECTIVE:**

We implement sequence and cluster analyses to generate new insights about exposure to pregnancy during the postpartum period using unique longitudinal data in a low-resource setting.

**METHODS:**

We used population-based data from a sample of 1,935 Ethiopian women who provided reports on factors influencing exposure to pregnancy in the year following childbirth. We used sequence and cluster analyses to characterize patterns of women’s reproductive behaviors during the postpartum period.

**RESULTS:**

We identified five postpartum trajectories of exposure to pregnancy: (1) no sex; (2) family-planning adopters, no menses; (3) family-planning adopters, return of menses; (4) sex, no menses, no family planning; and (5) sex, menses, no family planning. The ‘sex, no menses, no family planning’ cluster (50% of the sample) was characterized by resumption of sexual activity around three months postpartum, amenorrhea, and no contraceptive adoption. Women in the two ‘family-planning adopters’ clusters (39%) resumed sexual activity and adopted contraception around three months postpartum but differ by return of menses. The ‘no sex’ cluster (5%) was characterized by no sexual activity, contraceptive use, or menses.

**CONTRIBUTION:**

Sequence analysis offers new insights into a critical reproductive window by emphasizing the dynamic biological and behavioral states that influence distinct patterns of postpartum exposure to pregnancy. Establishing longitudinal trajectories of exposure to pregnancy has research and programmatic implications that include a more holistic understanding of postpartum fecundity and measuring unmet need for postpartum family planning.

## 1. Introduction

Although the World Health Organization (WHO) recommends postpartum family planning (PPFP) to promote healthy birth spacing (WHO 2013), and despite data suggesting that very few women (i.e., <10%) wish to become pregnant during the postpartum period (i.e., up to 12 months after birth), approximately 60% of postpartum women in low- and middle-income countries (LMICs) do not use contraception (Fotso et al. 2023; Karp et al. 2024; Moore et al. 2015; Pasha et al. 2015; Thiongo et al. 2022).

Low perceived exposure to pregnancy is an important yet understudied barrier to adopting contraception during the postpartum period. In addition to the reasons typically associated with nonuse of contraception (e.g., concerns about side effects and safety, limitations in access to preferred methods, and social, attitudinal, and couple barriers), women in the postpartum period have unique considerations surrounding perceived exposure of pregnancy (Chebet et al. 2015; Nakaggwa et al. 2023; Rossier et al. 2015; Tounkara et al. 2022; Tran et al. 2018). For example, women may accurately perceive that they are at low risk for conceiving if practicing the lactational amenorrhea method per guidance (Van der Wijden and Manion 2015) and choose to not use contraception during this period, but they may also overestimate protection if they deviate from exclusive, on-demand feeding or extend beyond six months (Cleland, Shah, and Benova 2015; Tran et al. 2018). Additionally, social norms in LMICs have historically encouraged periods of abstinence following birth for the health of the mother and child, rendering contraceptive use unnecessary (Alum et al. 2015; Desgrées-du-Loû and Brou 2005; Mbekenga et al. 2013; Mchome et al. 2020). Evidence suggests, however, that sexual initiation postbirth, particularly irregular sex, often occurs sooner than norms dictate; irregularity of intercourse may also contribute to inaccurate perceptions of exposure to pregnancy (Rossier et al. 2015).

Although studies on the unmet need for PPFP acknowledge the importance of considering changing exposure to pregnancy over the postpartum period (Cleland, Shah, and Benova 2015; Rossier et al. 2015), substantial knowledge gaps persist in understanding these dynamics. For example, limited and dated research has examined factors such as the timing of women’s resumption of sexual activity and return of the menstrual cycle during the postpartum period, as well as how these patterns vary across different contexts (e.g., factors related to cultural norms, patterns of breastfeeding, disease burden, diet) (Becker and Ahmed 2001; Borda, Winfrey, and McKaig 2010). Additionally, while there can be considerable variation in postpartum exposure to pregnancy within this relatively short time frame, few studies have used granular temporal data to effectively measure these dynamics. Addressing these gaps can improve our understanding of behavioral and biological aspects that underlie exposure to pregnancy, with the goal of ultimately enhancing the quality of counseling services. This understanding also aligns with the principles of person-centered contraceptive care and contraceptive autonomy, ensuring that women’s contraceptive choices are better attuned to their individual needs and preferences (Senderowicz 2020; Senderowicz et al. 2023).

The objective of this study is to use sequence and cluster analyses to generate new insights about exposure to pregnancy during the postpartum period using unique longitudinal data in an LMIC setting. We use monthly data on sexual activity, return of menses, adoption and discontinuation of contraception, and pregnancy to identify common postpartum trajectories and the biological and behavioral determinants that may increase or decrease women’s exposure to pregnancy and short birth intervals.

## 2. Study design

### 2.1 Setting and data

Ethiopia, the focus of our research, is a priority setting due to a high burden of short interpregnancy intervals and high maternal and newborn mortality; 47% of second or higher order births occur within an interpregnancy interval of less than 24 months. Most postpartum women in Ethiopia want to delay or limit childbearing during the first year (Karp et al. 2024). Further, despite 75% of women intending to use contraception in the immediate postpartum, PPFP use remains low; approximately 44% of women reported using any method of contraception at one year postpartum (Magalona et al. 2023).

We use data from the Performance Monitoring for Action (PMA) Ethiopia cohort study, a collaboration between the Johns Hopkins Bloomberg School of Public Health, Addis Ababa University, and the Ethiopian Federal Ministry of Health. PMA Ethiopia generates cross-sectional and longitudinal data on reproductive, maternal, and newborn health indicators to inform national and regional government priorities and policies. After completing a census of all households in the enumeration areas, including identification of currently pregnant and recently postpartum women, PMA Ethiopia enrolled a cohort of 2,879 pregnant/postpartum women and conducted a baseline survey, with follow-up surveys conducted at 6 weeks, 6 months, and 12 months postpartum. Data collection occurred from October 2019 to July 2021. Our analytic sample is restricted to women who completed the 12-month postpartum survey, given our desired observation period of 12 months, resulting in a sample size of *n* = 1,935 women, representing 71% of the original enrolled sample. PMA Ethiopia weights all data to account for study design, nonresponse, and attrition for the longitudinal panel data. More information about the study design of PMA Ethiopia can be found in Zimmerman et al. (2020).

We use information from several data elements in the 6- and 12-month surveys to generate a month-by-month account of each woman’s reproductive status for the 12 months following a live birth. This monthly information is derived from survey questions and a reproductive calendar that was administered to women at each follow-up survey. The reproductive calendar collects retrospectively reported monthly event data on a woman’s reproductive status each month since giving birth, namely whether the woman was using contraception, was pregnant, or gave birth. We combine this with additional data, including the month a woman reported resuming sexual activity and the month her menses returned. Both information points are crucial to understanding perceived and actual exposure to pregnancy but are not commonly included in postpartum studies (Brunie et al. 2013). Due to continuous survey data collection design and/or data collection errors, some women have months with missing statuses in their calendars (< 1% of the sample were missing the first through fourth calendar months). Following previous research, we allow women to have up to four months of missing data. Initially, we used the data elements to create 10 mutually exclusive monthly states, but given the low frequency of two states in exploratory analysis (‘no sex, menses, family planning (FP) use’ and ‘no sex, menses, no FP use’ each accounted < 1% of the sample for any given month), our final coding included women irrespective of menses resumption in the ‘no sex, FP use’ and ‘no sex, no FP use’ states. Given our interest in the exposure to pregnancy, we considered pregnancy an all-encompassing state. The final eight states included the following:

1. no sex, FP use
2. no sex, no FP use
3. sex, no menses, FP use
4. sex, menses, FP use
5. sex, no menses, no FP use
6. sex, menses, no FP use
7. pregnancy
8. missing

Of note, we did not include breastfeeding status because exclusive breastfeeding is notoriously hard to measure (Greiner 2014; WHO-UNICEF Technical Expert Advisory group on nutrition Monitoring (TEAM) 2018) and related to exposure to pregnancy in its relation to return of menses (Bongaarts 2015; Gray et al. 1990; Simpson-Herbert and Huffman 1981), for which we had direct questions. Further, the ubiquitous nature of breastfeeding in this population (over 90% of women reported breastfeeding at one year postpartum) reduced the likelihood that it would explain much variation in postpartum exposure to pregnancy.

### 2.2 Analytic approach: Sequence and cluster analysis

We conducted sequence and cluster analysis in R using the TraMineR (Gabadinho et al. 2011) and WeightedCluster (Studer 2013) packages to identify typical patterns of women’s 12-month postpartum reproductive behaviors and outcomes based on the eight monthly states. Sequence and cluster analyses are a set of data-driven tools used to describe and analyze categorical sequences characterizing longitudinal processes and are increasingly used in social sciences, life course research, and family demography (Barban and Sironi 2019; Liao et al. 2022). The benefits of these methods are their holistic approaches in considering the full trajectory of the observation period, allowing for the detection of typical timing, duration, and order of states subject to change. In contrast to time-based methods focusing only on timing (e.g., survival or event history analyses), sequence-based analysis allows for the characterization of timing, duration, and order of states subject to change. Combined with cluster analysis, researchers can identify typical longitudinal patterns among state sequences. These methods have been recently used to study demographic research questions such as dynamics of fertility intentions (Gemmill 2018), reproductive trajectories (Dejene and Gurmu 2022; MacQuarrie, Allen, and Gemmill 2021; MacQuarrie, Juan, and Gemmill 2022), childbearing trajectories (Bras, Remund, and Delaunay 2023; Bras and Schumacher 2019), and migration and fertility pathways (Delaporte and Kulu 2023). Yet to our knowledge, there have been no published studies using sequence and cluster analysis to examine postpartum trajectories of exposure to pregnancy.

We identify typical patterns in postpartum sequences using the following two steps. First, we used an algorithm to determine how similar each postpartum sequence is to all other sequences in the dataset by measuring distances, or differences, between pairs of sequences. There are many methods to calculate these dissimilarity measures, and decisions between appropriate methods are based on whether the research question is most interested in the timing, duration, or sequencing of trajectories (Studer and Ritschard 2016). Given our objective to identify the timing of when women become exposed to pregnancy, we opted to use a dynamic Hamming distance (DHD) method for our dissimilarity measure, which best preserves contemporaneous similarities between sequences. As the operations of insertion and deletion cause time warping, DHD uses only substitution cost to align sequences so that time shift will not occur (Lesnard 2010, 2014). In addition, to determine the distances between postpartum sequences, we utilized time-varying substitution costs (indelslog) as state changes can be more common at some time points than others. In the scenario of postpartum contraceptive use, changing from nonuse to use involves higher cost in early postpartum period than later in the trajectory (Lesnard 2010). We conducted sensitivity analyses using the DHD method with a substitution matrix based on transition rates, whose costs for the operations (e.g., insertion, deletion, and/or substitution) are data driven.

Second, we used the calculated dissimilarity matrix as input for the cluster analysis to identify typical patterns in postpartum sequences. Cluster analysis groups together women whose postpartum sequences exhibit similar patterns of the eight states as measured by the dissimilarity matrix using a partition around medoids (PAM) algorithm. This k-medoid algorithm searches for *k* representative sequences from the sample, or medoids, which are the individual sequences that have the smallest sum of distances to the sequences they represent in each cluster (Gemmill 2018; Liao et al. 2022). A sensitivity analysis using Ward’s clustering algorithm was also conducted. We used six quality metrics to evaluate the optimal number of clusters, along with discussion of the construct validity of each cluster among the authorship team (Gemmill 2018; Studer 2013): average silhouette width (weighted) (ASWw), Hubert’s gamma (HG), point biserial correlation (PBC), pseudo R2 (R2), pseudo R2-squared (R2sq), and Hubert’s C (HC). Higher scores indicate a more optimal model for all the quality metrics except HG, for which a lower score indicates a more optimal model. Once the final number of clusters were identified, we assigned descriptive names to each cluster of typical postpartum trajectories.

## 3. Results

### 3.1 Characteristics of the full sample

At baseline, women in the sample were around 27 (SE = 0.21) years of age, with most (83%) having at least one prior birth (Table 1). The majority had attended at least some primary school (59%), and over three-quarters lived in rural areas.

**Figure 1:**
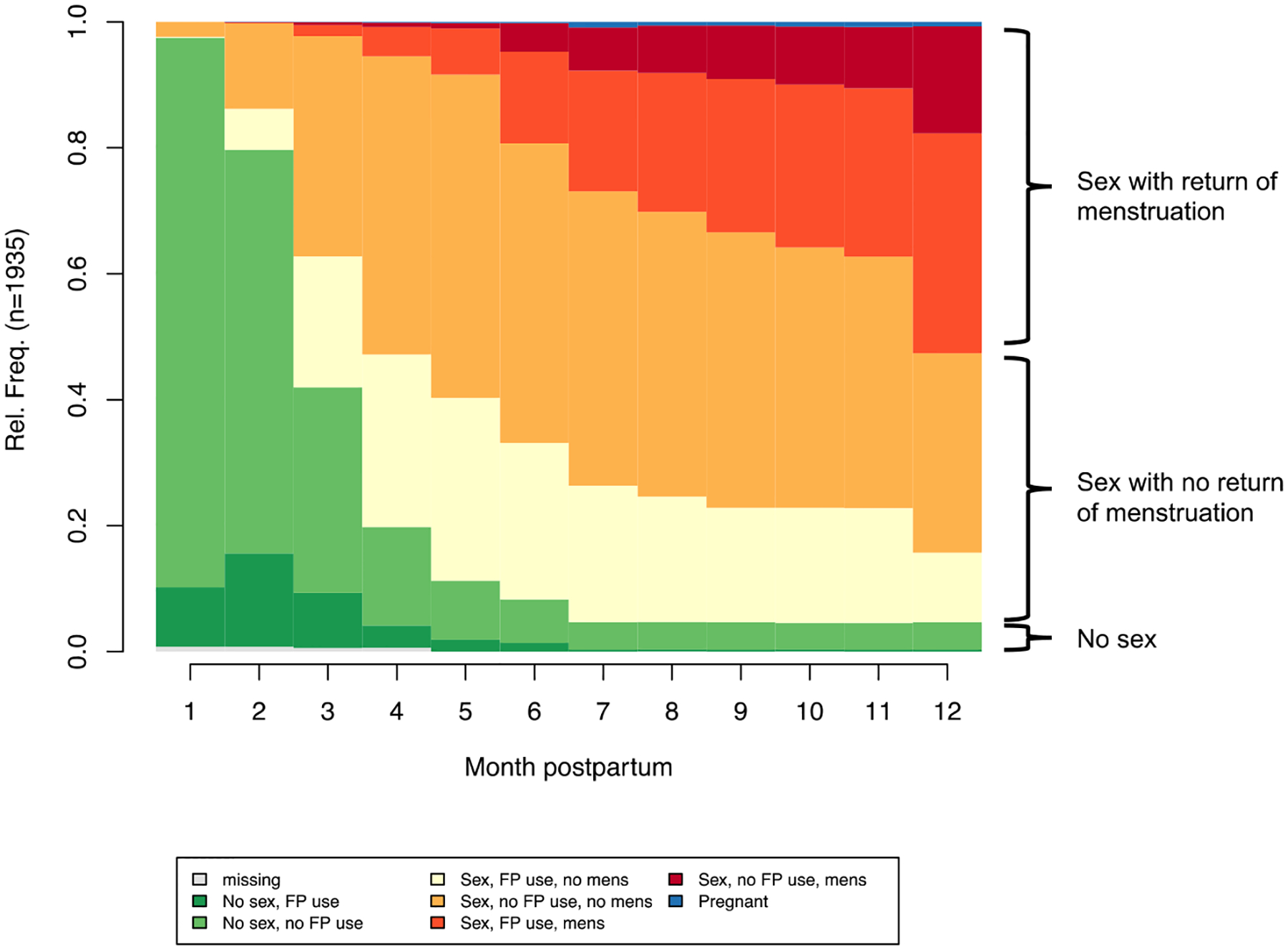
State distribution plot, Ethiopian postpartum women (*n* = 1,935), 2019–2021

Figure 1 presents the state distribution plot for the full sample and shows the percent of women in each state across the 12-month observation period, with corresponding data in Table 2. In the first month postpartum, almost all women report no sexual activity. By 2 months, about 20% of women are sexually active. By 6 months, most women (91%) have reported a return to sexual activity; among the 95% of women who return to sexual activity in the first year postpartum, the mean month of resumption is 3.5 (Table 2). Contraceptive use also increases over time; by 3 months postpartum, approximately one-third are using contraception. From 3 to 6 months, the percentage of women using contraception increases slightly, reaching about 40%, after which it increases to 48% by the end of the year. Women start to report the return of menses around 3 months postpartum; by 6 months, 17% of women’s menses had returned, and by 12 months, 49% report a return of menses (Table 2). Very few women report pregnancy (2%).

### 3.2 Typology of 12-month postpartum sequences

Figure 2 presents the quality metrics for selecting the optimal number of clusters using the DHD method with indelslog (Figure 2a) and transition rate (Figure 2b) substitution costs. Based on the criteria outlined in Studer (2013), we chose a five-cluster solution using the PAM approach.

**Figure 3:**
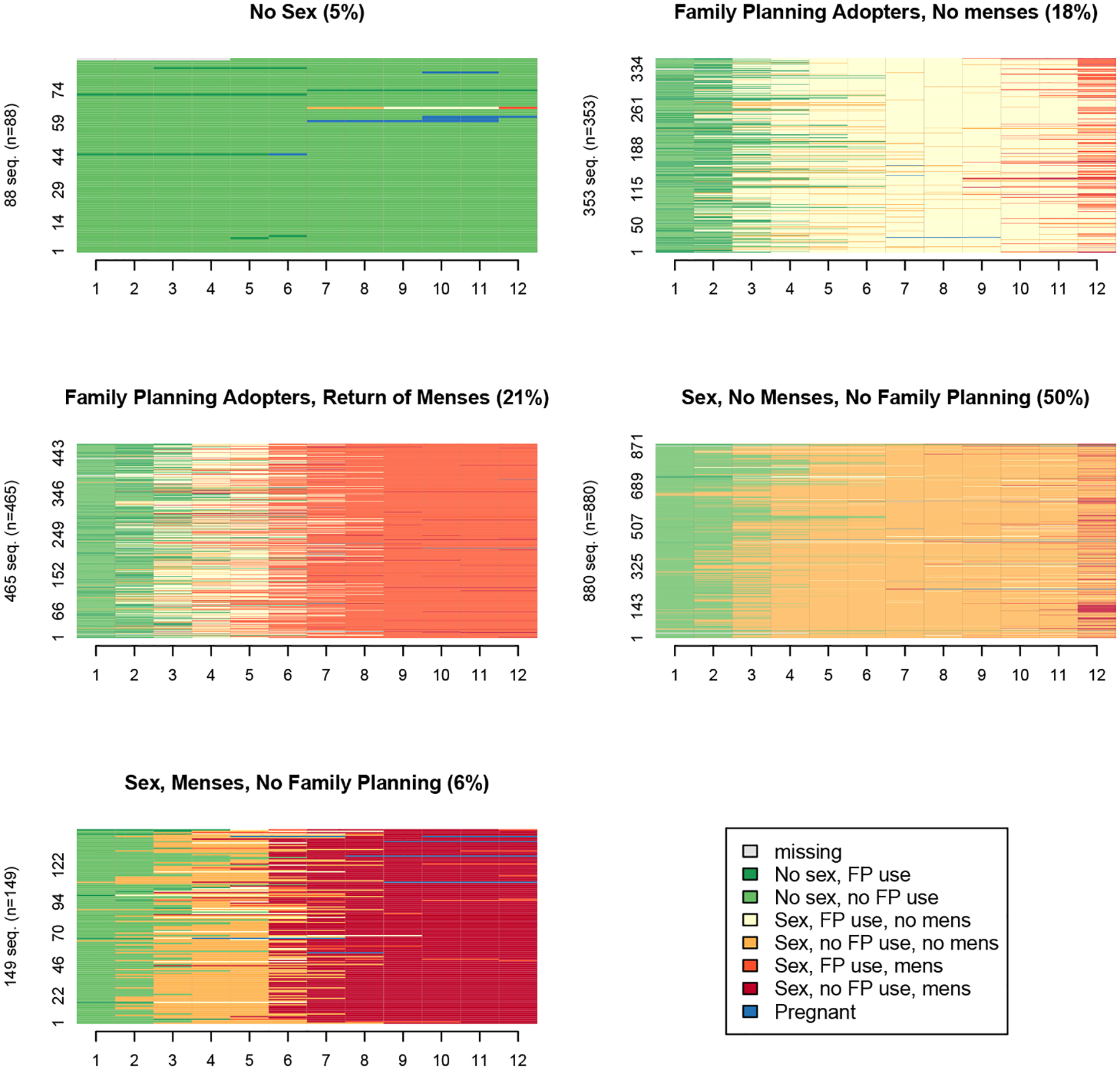
Sequence index plots for each cluster, Ethiopian postpartum women (*n* = 1,935), 2019–2021

Figure 3 presents sequence index plots, which show each woman’s full month-to-month sequence by cluster membership. For descriptive purposes, we assign clusters a label based on key features of each cluster: ‘no sex’; ‘family-planning adopters, no menses’; ‘family-planning adopters, return of menses’; ‘sex, no menses, no family planning’; and ‘sex, menses, no family planning.’ The most common cluster, ‘sex, no menses, no family planning,’ makes up half of the sample (50%) and is characterized largely by women who resume sexual activity around three months postpartum and do not report return of menses or contraceptive use. Still, approximately 17% of women in the ‘sex, no menses, no family planning’ cluster do report at least one month of use late in the postpartum period (Table 2). Women in the ‘sex, menses, no family planning’ cluster (6% of the sample) have similar trajectories to the ‘sex, no menses, no family planning’ group in the timing of resumption of sexual activity and low use of contraception, yet most report menses returning around six months. The sequence index plot for this cluster also reveals that contraceptive use was reported at some point postpartum by 30% of women (Table 2), but the duration of use can be visually seen as brief and followed by nonuse. While pregnancy was not a common enough state to merit its own cluster, it is visually most prevalent in the ‘sex, menses, no family planning’ cluster, where 4% of women reported a pregnancy.

Women belonging to the second largest cluster (21%), the ‘family-planning adopters, return of menses’ group, return to sexual activity around three months postpartum, but they also adopt contraception around this month. These women report their menses returning around six months. The third largest cluster (18%), ‘family-planning adopters, no menses’ group, are similar to the ‘family-planning adopters, return of menses,’ except they report menses returning later or not at all during the 12-month period of observation. Finally, the last cluster, ‘no sex,’ accounts for 5% of the sample. These women largely report no sexual activity nor contraceptive use, though we can see a handful of contraceptive adoption and pregnancy experiences occurring later in the trajectory. While it may seem implausible to have women in this cluster experiencing pregnancy, sequence analysis groups together trajectories that are most similar, not identical; therefore, women experiencing pregnancy later in their trajectories could still be more similar to the other women in this cluster given their no sex, nonuse status of the months prior to the onset of pregnancy. The transition from no sex/nonuse to pregnancy could be possible by becoming pregnant in the first month’s return to sexual activity.

## 4. Conclusion

We used sequence and cluster analysis to identify common trajectories of exposure to pregnancy among a cohort of postpartum Ethiopian women, marking a novel use of this method to study the postpartum period. Our findings underscore the significance of timing and sequencing in shaping the overall trajectory of exposure to pregnancy among a population where most women do not desire to become pregnant and therefore may be at risk of unintended pregnancy. Our approach is advantageous in condensing a period of immense transitions between states with dynamic exposure to postpartum pregnancy, while also considering nuanced trajectories that cannot be captured in analyses that focus solely on transitions to a singular event.

Our results contribute to demographic scholarship on fertility and place renewed emphasis on understanding biological and behavioral factors that influence women’s fecundity postpartum, a dimension that is often overlooked in the literature. It is perhaps surprising that half of our sample belonged to the ‘sex, no menses, no family planning’ group, where the potential for unintended pregnancy among these women remains debatable (Rossier et al. 2015). From a programmatic perspective, women are advised to proactively use contraception to prevent pregnancy given that ovulation can occur before a first observed menstruation. However, from a demographic perspective, this finding may indicate lower population fecundity in postpartum Ethiopian women, given the low reported incidence of pregnancy. Variation in the duration of postpartum amenorrhea between populations is an understudied phenomenon, likely driven by biosocial factors, such as the intensity and duration of breastfeeding and nutritional status, which are additionally influenced by gender and socioeconomic power relations (Ersino et al. 2018; Eslami et al. 1990; Khalil et al. 1996; Lewis et al. 1991; Todd and Lerch 2021; Wanjohi et al. 2017). Further research in this area is essential to deepen our understanding.

This work also has implications for PPFP research, counseling, and programs. From a research perspective, our findings provide important context for debates around measuring unmet needs for PPFP (Cleland, Shah, and Benova 2015; Gahungu, Vahdaninia, and Regmi 2021; Rossier et al. 2015), particularly by elucidating dynamic factors that shape exposure, including timing of resumption of sexual activity and prolonged amenorrhea, both of which may under- or overestimate needs. These insights also have implications for PPFP counseling for similar reasons and may influence appropriate timing of services.

Our study has several limitations. First, the data we use do not include information on other factors that could influence exposure to pregnancy, including detailed measures of hormonal levels and nutritional markers. Relatedly, our measure of sexual activity was not collected every month; instead, we used the reported month of return to sexual activity as a proxy for sexual activity throughout the period, but there could certainly be differential variation in the frequency of sexual activity. Second, our study relied on retrospective reproductive calendar data, which has the potential for data quality issues. Of note, our incidence of pregnancy may be underestimated due to underreporting of pregnancies in early gestation, miscarriages, or abortions (MacQuarrie et al. 2018). Other known data quality issues include heaping (Bradley, Winfrey, and Croft 2015) and reduced reliability due to recall bias, particularly for short-acting methods (Anglewicz et al. 2023). Third, our results are not generalizable to other settings, which could differ in terms of resumption of sexual activity, return of menses, postpartum adoption, or other unobserved factors. Fourth, there is some subjectivity in the evaluation of metrics and clustering decisions in our approach. We conducted several sensitivity analyses to evaluate whether our conclusions were robust to different choices. Finally, the PMA Ethiopia study overall has a 16% attrition rate, which is considered standard for panel surveys in LMICs (Anglewicz et al. 2023; Karp et al. 2024; Odwe et al. 2021). Panel weights account for differential loss to follow up by age, education, marital status, wealth, and residence at baseline, yet there may be unobserved differences in those retained in the panel. Additionally, data collection began in Tigray in 2019 prior to the onset of civil conflict, circumstances that contributed to higher than anticipated loss to follow up.

This descriptive finding serves as a proof of concept for the applicability of sequence and cluster analysis in examining reproductive dynamics by offering a nuanced understanding of how trajectories unfold over time, emphasizing processes from a holistic perspective. Our results pave the way for future research to use the output to explore the multilevel correlates or predictors of group membership. Such analyses could incorporate individual factors, like sociodemographic characteristics, as well as broader community or health system influences, such as access to family-planning services. Integrating multilevel determinants will provide deeper insights into the drivers of postpartum exposure to pregnancy and how these vary within and across populations.

## Figures and Tables

**Figure 2a:**
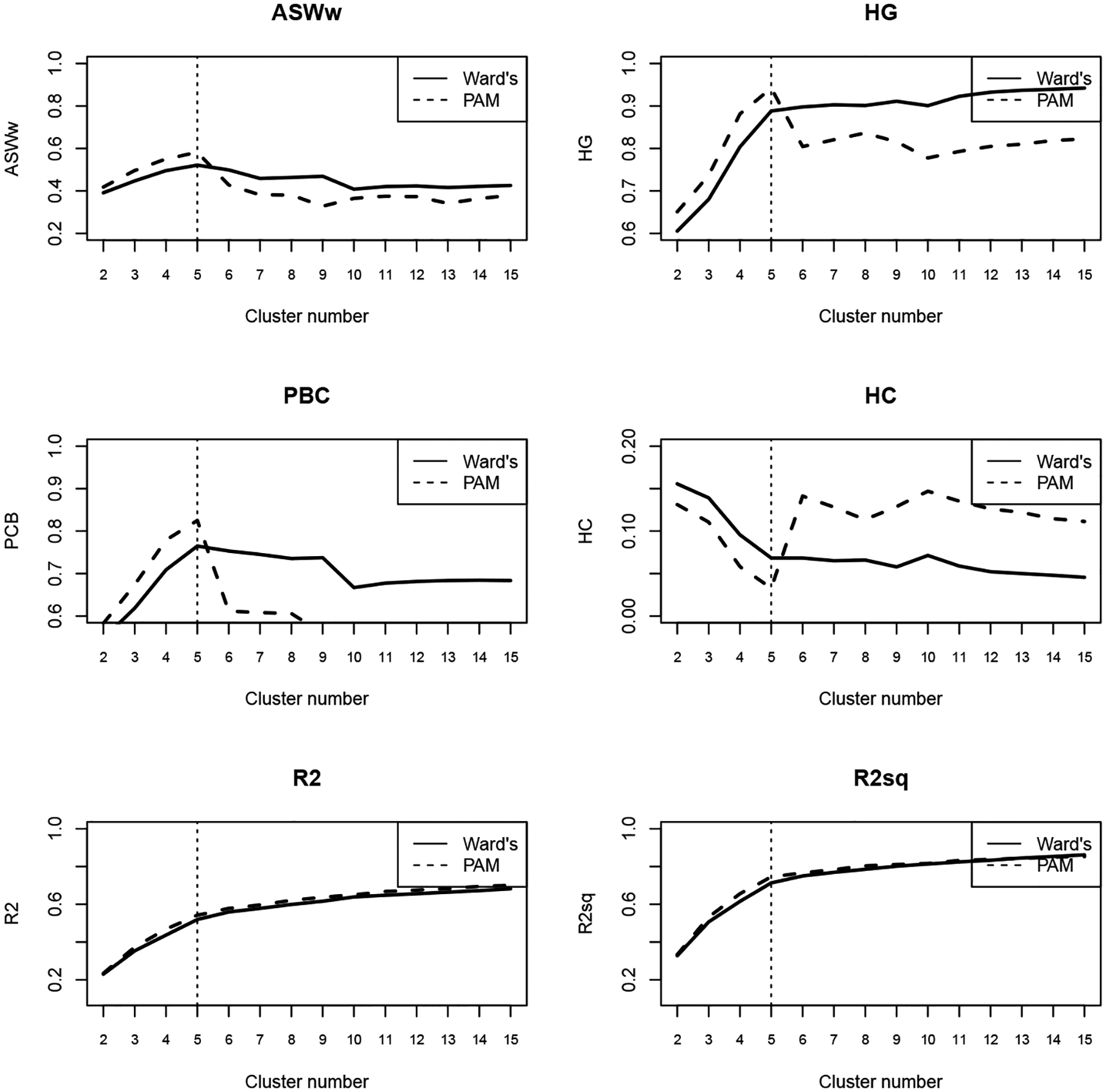
Metrics with dynamic Hamming distance and time-varying cost matrix (indelslog)

**Figure 2b:**
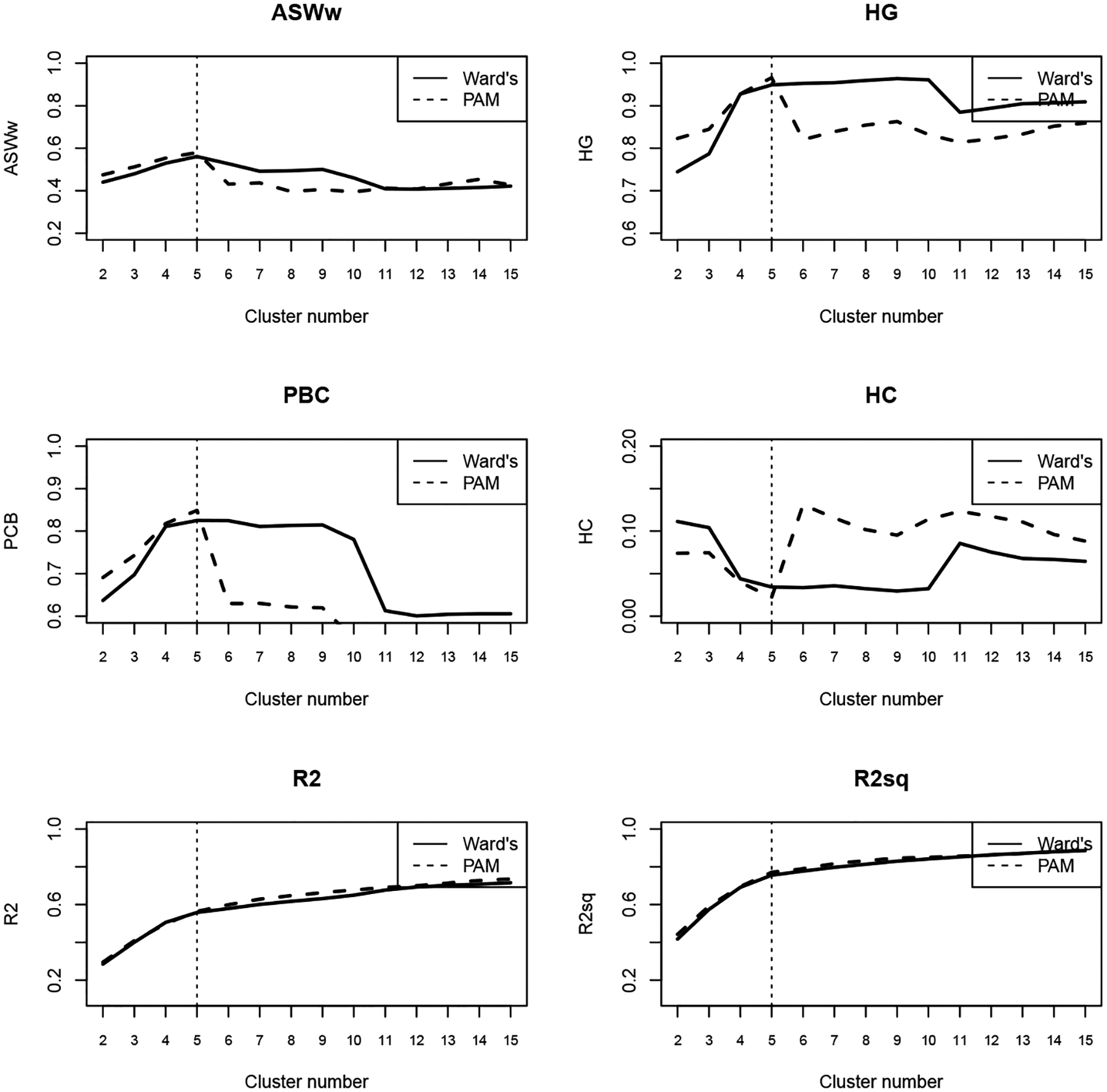
Metrics with dynamic Hamming distance and transition rate cost matrix

**Table 1:** Sociodemographic characteristics of the full sample (*n* = 1,935), Ethiopian postpartum women, 2019–2021

Sociodemographics (measured at baseline)		Full sample, *n* = 1,935
Age	Mean years (SE)	27.21 (0.21)
Parity	0 children	17
	1–2 children	38
	3+ children	45
Highest level of education		
	None	41
	Primary	40
	Secondary	19
Residence	Urban	22
	Rural	78
Wealth	Lowest quintiles	40
	Higher quintiles	60

**Table 2:** Trajectory characteristics for the full sample and five clusters, Ethiopian postpartum women, 2019–2021

		No sex	Family-planning adopters, no menses	Family-planning adopters, return of menses	Sex, no menses, no family planning	Sex, menses, no family planning
Row %	100%	5	18	21	50	6
**Trajectory characteristics**						
% return to sexual activity	95	1	99	100	100	100
Month of return to sexual activity	3.5	7.0	3.4	3.4	3.5	3.4
% menses returned	49	46	37	100	26	100
Month of return of menses	8.3	9.3	11.4	6.0	11.3	6.5
% contraceptive adoption	48	6	96	98	17	30
Month of adoption	4.2	4.2	3.3	3.4	8.0	5.0
% pregnant	2	6	1	3	1	4
